# Cortical Synaptic Transmission and Plasticity in Acute Liver Failure Are Decreased by Presynaptic Events

**DOI:** 10.1007/s12035-016-0367-4

**Published:** 2017-01-23

**Authors:** Mariusz Popek, Bartosz Bobula, Joanna Sowa, Grzegorz Hess, Rafał Polowy, Robert Kuba Filipkowski, Małgorzata Frontczak-Baniewicz, Barbara Zabłocka, Jan Albrecht, Magdalena Zielińska

**Affiliations:** 1 Department of Neurotoxicology, Mossakowski Medical Research Centre, Polish Academy of Sciences, Pawińskiego 5 St, 02-106 Warsaw, Poland; 2 Department of Physiology, Institute of Pharmacology, Polish Academy of Sciences, Smętna 12 St, 31-343 Cracow, Poland; 3 Behavior and Metabolism Research Laboratory, Mossakowski Medical Research Centre, Polish Academy of Sciences, Pawińskiego 5 St, 02-106 Warsaw, Poland; 4 Electron Microscopy Platform, Mossakowski Medical Research Centre Polish Academy of Sciences, Pawińskiego 5 St, 02-106 Warsaw, Poland; 5 Molecular Biology Unit, Mossakowski Medical Research Centre, Polish Academy of Sciences, Pawińskiego 5 St, 02-106 Warsaw, Poland

**Keywords:** Acute liver failure, Presynaptic events, Neurotransmission

## Abstract

Neurological symptoms of acute liver failure (ALF) reflect decreased excitatory transmission, but the status of ALF-affected excitatory synapse has not been characterized in detail. We studied the effects of ALF in mouse on synaptic transmission and plasticity ex vivo and its relation to distribution of (i) synaptic vesicles (sv) and (ii) functional synaptic proteins within the synapse. ALF-competent neurological and biochemical changes were induced in mice with azoxymethane (AOM). Electrophysiological characteristics (long-term potentiation, whole-cell recording) as well as synapse ultrastructure were evaluated in the cerebral cortex. Also, sv were quantified in the presynaptic zone by electron microscopy. Finally, presynaptic proteins in the membrane-enriched (P2) and cytosolic (S2) fractions of cortical homogenates were quantitated by Western blot. Slices derived from symptomatic AOM mice presented a set of electrophysiological correlates of impaired transmitter release including decreased field potentials (FPs), increased paired-pulse facilitation (PPF), and decreased frequency of spontaneous and miniature excitatory postsynaptic currents (sEPSCs/mEPSCs) accompanied by reduction of the spontaneous transmitter release-driving protein, vti1A. Additionally, an increased number of sv per synapse and a decrease of P2 content and/or P2/S2 ratio for sv-associated proteins, i.e. synaptophysin, synaptotagmin, and Munc18–1, were found, in spite of decreased content of the sv-docking protein, syntaxin-1. Slices from AOM-treated asymptomatic mice showed impaired long-term potentiation (LTP) and increased PPF but no changes in transmitter release or presynaptic protein composition. Our findings demonstrate that a decrease of synaptic transmission in symptomatic ALF is associated with inefficient recruitment of sv proteins and/or impaired sv trafficking to transmitter release sites.

## Introduction

**Fig. 1:**
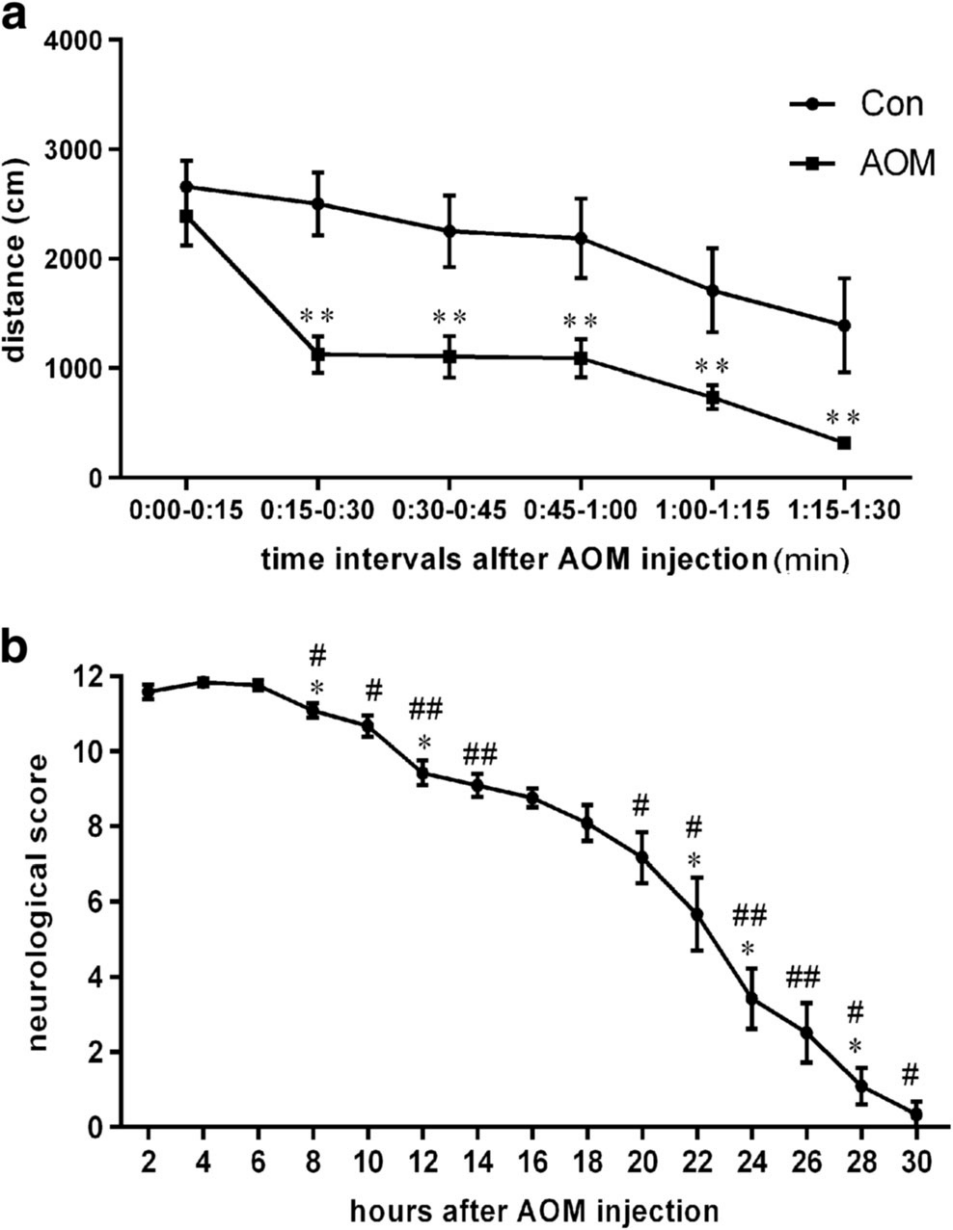
Novel-cage activity and neurological assessment. **a** Shorter distance traveled by AOM-injected mice shortly after placing into a new cage; **p < 0.01, *n* = 9. **b** Decreased neurological score following AOM injection. *Asterisk* indicates *p* < 0.05 vs. the results of the previous session (2 h earlier), and hashtag indicates *p* < 0.05 (*p* < 0.01) vs. 4-h-earlier session; *n* = 12. Results are means ± SEM

**Fig. 2:**
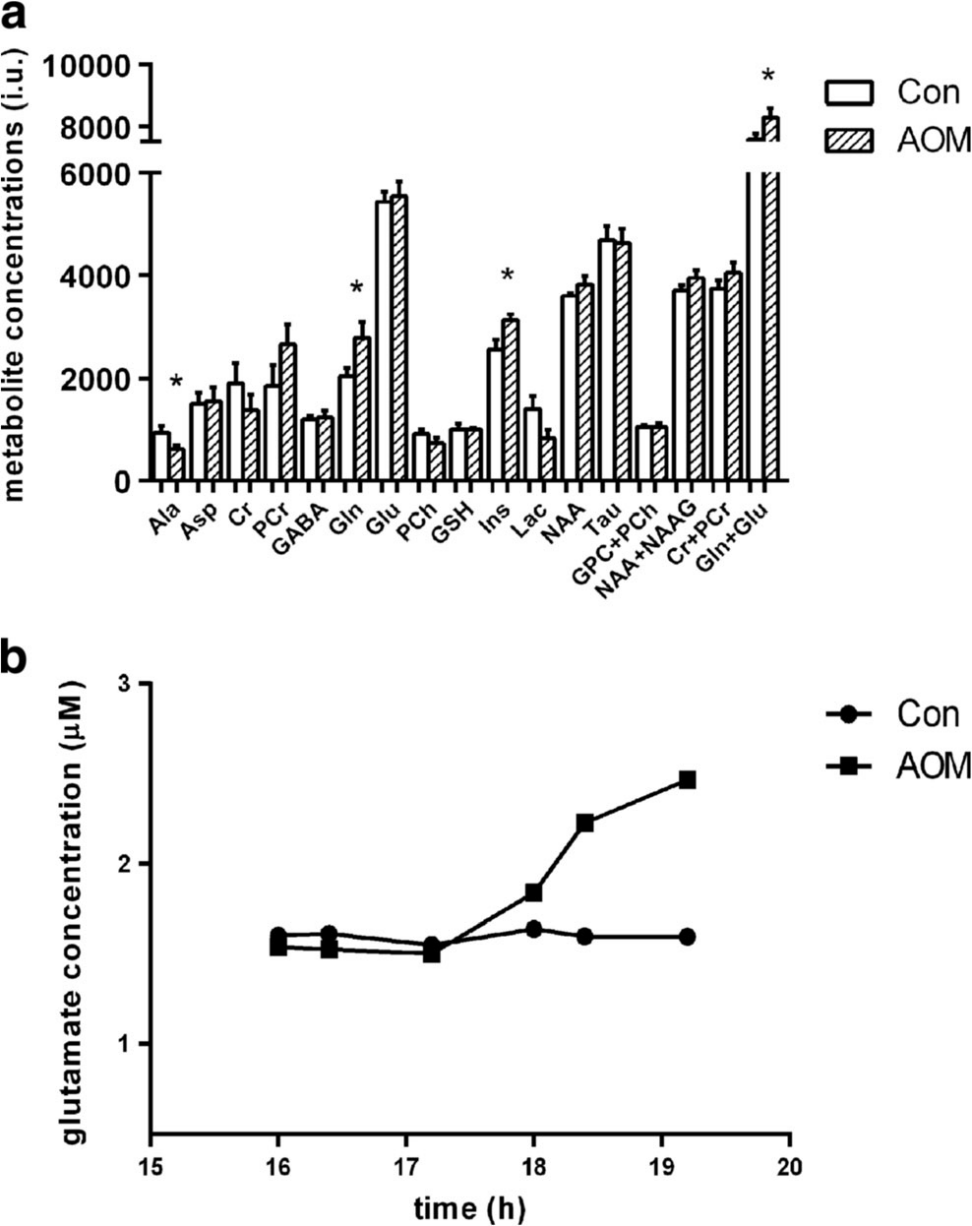
Metabolite analysis by ^1^H magnetic resonance spectroscopy technique and cerebral cortex microdialysis of AOM mice. **a** Spectrometric analysis revealed ALF-specific changes in cerebral cortex of AOM mice. *Asterisk* indicates *p* < 0.05 vs. Con; *n* = 12. Analyzed metabolites *Ala* alanine, *Asp* aspartic acid, *Cr* creatine, *PCr* phosphocreatine, *GABA* gamma-aminobutyric acid, *Gln* glutamine, *Glu* glutamate, *PCh* phosphocholine, *GSH* glutathione, *INS myo*-inositol, *Lac* lactate, *NAA* N-acetylaspartate, *Tau* taurine, *GPC* glycerophosphocholine, *NAAG* N-acetylaspartylglutamate. **b** Extracellular glutamate concentration, as determined in microdialysates from freely moving mice, was significantly elevated after AOM administration; *n* = 4. Results are means ± SEM

Neuropsychiatric symptoms of acute or chronic liver failure (ALF or CLF), collectively defined as hepatic encephalopathy (HE), are associated with a decline of excitatory neurotransmission [1–3]. Present knowledge of the electrophysiological and molecular correlates of ALF- or CLF-affected synapse is confined mostly to the postsynaptic events related to altered Glu receptor-mediated signaling pathways [4] and/or synaptic plasticity, i.e., long-term potentiation (LTP) and LTD [5], while the status of the presynaptic region involved in neurotransmitter release has remained unattended. A few earlier reports have dealt with alterations in the synaptic events in brain preparations treated with ammonia, which is a major neurotoxin implicated in HE [6], emphasizing the role of postsynaptic changes [7–10]. However, the observations were confined to short-term effects (seconds to minutes), which cannot be considered representative for the progression of ALF symptoms lasting for hours to weeks. In this study, therefore, we address the question whether and to what extent ALF disturbs the functional and structural integrity of the presynaptic neurotransmitter release machinery in addition to postsynaptic changes. We therefore measured expression and distribution of presynaptic proteins including (i) the integral vesicle proteins synaptophysin and synaptotagmin; (ii) a member of the Sec1/Munc18-like protein family Munc-18-1; (iii) an integrated SNARE protein syntaxin-1; and (iv) vt1 interactor 1a (vti1a) protein, which characterizes vesicles driving spontaneous release [11–13]. The response of presynaptic proteins to ALF was compared to that of the constituents of the postsynaptic complex active in signal transduction, NMDA subunit NR1, PSD95, and the neuronal form of nitric oxide synthase (nNOS). The analysis was carried out in mice in which ALF was induced with a hepatotoxin, azoxymethane (AOM), and was preceded by thorough behavioral, biochemical, and neurophysiological characterization of the model. The results presented in Table 1 and Figs. 1 and 2 confirm and extend the findings by others that the mouse AOM model is a valid model of acute HE in humans [14–16].

**Table 1:** Increased concentration of cytokines, ammonia, and activity of liver damage marker enzymes in serum of control and AOM mice

	Con	AOM
IL-6 (pg/ml)	10.0 ± 2.2	214.5 ± 34.5*
TNF-α (pg/ml)	3.1 ± 0.2	14.7 ± 1.8*
AST (IU/I)	172.4 ± 30.6	805.4 ± 92.2*
ALT (IU/I)	46.5 ± 6.1	559.0 ± 52.8*
Ammonia (mg/ml)	0.0052 ± 0.0011	0.0140 ± 0.0010*

*p < 0.05 vs. Con; *n* = 6

Cerebral cortical slices derived from AOM-treated asymptomatic and symptomatic mice were subjected to the electrophysiological analysis of field potentials (FPs), including paired-pulse facilitation (PPF) ratio, parameters characterizing excitability of pyramidal neurons, and spontaneous excitatory postsynaptic currents (sEPSCs) as well as miniature excitatory postsynaptic currents (mEPSCs).

Growing evidence that liver failure affects synaptic plasticity and that Glu release plays a crucial role in driving the postsynaptic events [17] prompted us to assess long-lasting activity-dependent changes in synaptic efficiency, known as LTP. LTP underlies multiple forms of synaptic plasticity in the brain [18]. Analysis of the morphological and biochemical status of the synapse focused on the neurotransmitter release apparatus. Ultrastructural assessment of the presynaptic zone comprised the size and content of sv. Next, we examined the content and distribution between the cytosolic- and membrane-enriched compartments of the cerebral frontal cortex of the abovementioned representative presynaptic and postsynaptic proteins.

## Material and Methods

### AOM Model of Acute Liver Failure in Mice

All experiments were performed with agreement of local animal ethical committee in Warsaw in accordance with EC Directive 86/609/EEC. Male C57BL/6 mice (animal colony of the Mossakowski Medical Research Centre, Polish Academy of Sciences in Warsaw), body weight 30.0 ± 5.1 g, were subjected to a hepatotoxic insult by single, intraperitoneal AOM injection at 100 mg/kg b.w. The mice had free access to water and chow and were housed in constant temperature, humidity, and 12-h light-dark cycling. If not otherwise stated, experiments were performed in the asymptomatic stage, 4 h after AOM injection, and symptomatic stage, 18 h after AOM injection. The selection of the time points was based on the neurological performance of mice.

### Activity Assessment

For activity assessment, immediately after AOM (*n* = 11) or saline (*n* = 5) injections, the mice were placed individually in novel cages (43 × 27 × 15 cm), with fresh bedding, covered by a metal 1 × 1-cm grid to allow observation from above. The experiment room was illuminated by dim red light. The animals were recorded by an infrared acA1300-60NIR camera (Bassler AG, Germany) for 4 h, and the activity was measured using Ethovision XT 10 (Noldus Information Technology, Netherlands).

### Neurological Assessment

Neurological decline was determined in a group of mice (*n* = 12) by measuring the corneal reflex, pinna reflex, vibrissae reflex, startle reflex, righting reflex, and postural reflex [16, 19], with each given a score of 0 (no reflex evident), 1 (weak or delayed reflex), or 2 (regular reflex), resulting in a neurological score in the range of 0 to 12. The reflexes were measured every 2 h after AOM injection. Corneal reflex was assessed by touching the eye with saline-soaked cotton applicator and observing a blink response [14, 20]. To determine pinna reflex, the earlobe was touched with a cotton rod and ear retraction was observed [20, 21]. To assess vibrissae response, the whiskers were brushed and reactional head movement was observed [21, 22]. Startle reflex was tested by presenting a sudden, unexpected noise and evaluating the reaction of the animal [15, 23]. Righting reflex was determined by placing the mice on their backs and assessing how fast they right themselves [20, 24]. Postural reflex was tested by placing the mice individually in a cage without bedding and rapidly moving it in cardinal directions and judging how the animals try to keep their balance [20, 25].

### Biochemical Blood Analyses

#### Enzymatic Determination of Liver Enzymes and Ammonia in Serum

Blood was collected into Eppendorf and after the formation of a clot, centrifuged at 8000×*g* for 6 min. Then using enzymatic tests for ammonia, alanine aminotransferase (ALT) and aspartate aminotransferase (AST), the absorbance were measured at a wavelength of 340 nm after mixing probe with reagent and every 60 s for 2 min in 37 °C. Using an appropriate model, based on the difference in absorbance in time (Δabs/min), results are expressed as IU/l.

#### Cytokine IL-6 and TNF-α Concentrations in Serum

Blood was collected into Eppendorf and after the formation of a clot, centrifuged at 6500×*g* for 10 min. The microsphere-based immunoassay (cytometric bead array (CBA); BD Biosciences) was performed in accordance to the commercial protocol with modifications [26]. Briefly, six microsphere populations with distinct fluorescence intensities were dyed with proprietary dyes (emission 650 nm). These beads were coated with capture antibodies against cytokines and mixed with recombinant standards or serum and the phycoerythrin (PE)-conjugated cytokine antibodies (emission 585 nm) to form sandwich complexes. The instrument setup was performed with CaliBRITE beads, and the cytometer setup beads (BD) were done according to the manufacturer’s instructions; 2000 events were measured and analyzed. A monomeric microsphere population was gated on forward and side scatters. Data were analyzed in two-color fluorescence dot plots representing the different microsphere populations (emission 650 nm) and the cytokine concentration (according to PE emission 585 nm). Mean fluorescence intensity values were collected. Four-parametric logistic calibration curves were used, and results were expressed as pg/ml.

### Metabolite Analysis by ^1^H Magnetic Resonance Spectroscopy Technique and Cerebral Cortex Microdialysis of Symptomatic AOM Mice

#### ^1^H Magnetic Resonance Spectroscopy Acquisition and Processing

To obtain the spectra of brain metabolites, localized proton spectroscopy (Biospec 70/30USR) at short echo was performed using PRESS sequence (TR/TE = 2000/20 ms, 512 averages, 2048 points, scan time = 17 min) with VAPOR water suppression, the outer volume suppression, and frequency drift correction (flip angle 5°). Eddy current correction was performed at the scanner. Each measurement was carried out in two separated volumes of interest (VOI). The VOI (4 × 2 × 1.5 mm^3^) encompassed the frontal cortex. Linear and second-order global shims were adjusted with ADJ_1st_2nd_order protocol. Afterwards, linear and second-order local shims were automatically adjusted with FASTMAP in a cubic volume which contained the volume of interest region (4 × 4 × 4 mm^3^ for the frontal cortex). The unsuppressed water line width was typically maintained at around 9–12 Hz.

Metabolite concentrations were determined using a linear combination analysis method LCModel [27] (http://www.s-provencher.com/pages/lcmodel.shtml). The unsuppressed water signal measured from the same volume of interest was used as internal reference for absolute metabolite quantification. Metabolite concentrations are reported in institutional unit (IU). The spectrum signal to noise ratio was typically at around 12–25.

#### Microdialysis on Freely Moving Mice

Mice were anesthetized with isoflurane (3.5% in air), placed in a stereotaxic frame, and kept asleep at a flow rate of 1.5%. The head was shaved and decontaminated by 70% ethanol, and two 1.2-mm-diameter holes were drilled, first at coordinates AP −0.5, ML +0.5, in which skull screw was inserted, and second at coordinates AP +2.0, ML −0.5, DV −0.5 according to the atlas [28], in which a guide cannula was inserted. Guide cannula was implanted at a depth of 0.5 mm. Skull surface and guide cannula were secured with dental acrylic cement. Before arousals, antibiotic (Baytril, 2.5%; 0.2 ml/kg b.w.) and a painkiller (Ketonal, 2.5 mg/kg b.w.) were subcutaneously administered. Animals were placed in 21-cm-diameter cylindrical cages with ad libitum access to food and water. One day after surgery, mice were anesthetized with isoflurane (3.5% in air) and a probe was implanted. After preparing the probe in accordance with the instructions, ACSF of the following composition (in mM): NaCl (130), KCl (5), CaCl_2_ (2.5), MgSO4 (1.3), KH_2_PO_4_ (1.25), NaHCO_3_ (26), and D-glucose (10), bubbled with a mixture of 95% O_2_ and 5% CO_2_ passes through it. After 1 h in 2.5-μl/h flow, samples were collected every 40 min (100 μl) for 4 h (six fractions). After this, AOM was i.p. injected, and after 16 h, samples were collected every 40 min. All samples were immediately frozen at −80 °C.

#### High-Performance Liquid Chromatography Determination of Glutamate

Glutamate concentration in mice microdialysates was measured using HPLC with fluorescence detection after derivatization in a timed reaction with o-phthalaldehyde plus mercaptoethanol, exactly as described earlier [29].

#### Electrophysiological Studies

The brains were rapidly removed from the skulls and immersed in an ice-cold ACSF of the following composition (in mM): NaCl (130), KCl (5), CaCl_2_ (2.5), MgSO_4_ (1.3), KH_2_PO_4_ (1.25), NaHCO_3_ (26), and D-glucose (10), bubbled with a mixture of 95% O_2_ and 5% CO_2_. Frontal cortical slices, 400 μm thick, were cut in the coronal plane using a vibrating microtome (Leica). Slices were stored at 32.0 ± 0.5 °C.

#### FP Recording and LTP Induction

Individual slices were placed in the recording chamber of an interface type which was superfused (2.5 ml/min) with a modified ACSF (temperature 32.0 ± 0.5 °C) containing (in mM) NaCl (132), KCl (2), CaCl_2_ (2.5), MgSO_4_ (1.3), KH_2_PO_4_ (1.25), NaHCO_3_ (26), and D-glucose (10), bubbled with 95% O_2_ and 5% CO_2_ (temperature 32.0 ± 0.5 °C). A concentric bipolar stimulating electrode (FHC, USA) was placed in cortical layer V. Stimuli of 0.033-Hz frequency and duration of 0.2 ms were applied using a constant-current stimulus isolation unit (WPI). Glass micropipettes filled with ACSF (2–5 MΩ) were used to record field potentials. Recording microelectrodes were placed in cortical layer II/III. The responses were amplified (EXT 10–2 F amplifier, NPI), filtered (1 Hz–1 kHz), A/D converted (10-kHz sampling rate), and stored on PC using the Micro1401 interface and Signal 2 software (CED). A stimulus-response (input-output) curve was made for each slice. To obtain the curve, stimulation intensity was gradually increased stepwise (15 steps; 5–100 μA). One response was recorded at each stimulation intensity. Then, stimulation intensity was adjusted to evoke responses of 30% of the maximum amplitude. LTP was induced by theta burst stimulation (TBS). TBS consisted of 10 trains of stimuli at 5 Hz, repeated 5 times every 15 s. Each train was composed of five pulses at 100 Hz. During TBS, pulse duration was increased to 0.3 ms.

#### Whole-Cell Recording

Individual slices were placed in the recording chamber mounted on the stage of the Axioskop (Zeiss) microscope and superfused at 2 ml/min with ACSF. Recording pipettes were pulled from borosilicate glass capillaries (Harvard Apparatus) using Sutter Instrument P97 puller. The pipette solution contained (in mM) 130 K-gluconate, 5 NaCl, 0.3 CaCl_2_, 2 MgCl_2_, 10 HEPES, 5 Na2-ATP, 0.4 Na-GTP, and 1 EGTA (osmolality 290 mOsm, pH = 7.2). Pipettes had open tip resistance of approximately 6 MΩ. Pyramidal cells were sampled from the sites located approximately 2 mm lateral to the midline and approximately 0.3 mm below the pial surface. Signals were recorded using the SEC 05LX amplifier (NPI), filtered at 2 kHz, and digitized at 20 kHz using Digidata 1440A interface and Clampex 10 software (Molecular Devices).

After obtaining the whole-cell configuration and subsequent 10-min stabilization period, the firing characteristics of the recorded cells were assessed using intracellular injections of rectangular current pulses of increasing amplitude (duration 400 ms) in the current clamp mode. For each cell, the relationship between injected current intensity and the number of spikes was plotted. The gain was determined as a slope of the straight line fitted to experimental data. The threshold current (*I*_th_) was determined as a current extrapolated at zero firing rate. To record sEPSCs, neurons were voltage clamped at −76 mV and synaptic events were recorded for 4 min as inward currents. To record mEPSCs in some experiments, 1 μM tetrodotoxin (TTX) was added to the ACSF spontaneous and miniature EPSCs were detected offline using the automatic detection protocol (Mini Analysis software, Synaptosoft Inc.) and subsequently checked manually for accuracy. Data were accepted for the analysis when the access resistance ranged between 15 and 18 MΩ and it was stable (<25% change) during recording. The threshold amplitude for the detection of an EPSC was set at 5 pA.

#### Transmission Electron Microscopy

The animals were anesthetized and perfused through the ascending aorta with 2% paraformaldehyde and 2.5% glutaraldehyde in 0.1 M cacodylate buffer, pH 7.4. The cerebral cortex was fixed in the same solution for 20 h (at 4 °C) and placed in a mixture of 1% OsO_4_ and 0.8% K_4_[Fe(CN)_6_]. Electron microscopy analysis was performed on 10 sections from each experimental and control animals. Synapses were classified according to their morphology. For the analysis, only asymmetric (i.e., excitatory) synapses were taken into account. Sv were quantified by counting them in all visible synapses at each section (in all fields). Sv were counted in specimens from control and symptomatic AOM animals. The average number of the vesicles was calculated for each group.

#### Immunoblotting Analyses

Immediately removed mice brain cortex was isolated on ice, homogenized in buffer (20 mM Tris-HCl, pH 6.8; 137 mM NaCl; 2 mM EDTA; 1% Triton X-100; 0.5 mM DTT; 0.5 mM PMSF; Phosphatase Inhibitor cocktail 1:100; Protease Inhibitor Cocktail 1:200), and centrifuged at 12000×*g* for 10 min. To separate P2 and S2 fractions, cortex was homogenized in buffer (15 mM Tris-HCl, pH 7.6; 0.25 M sucrose, 1 mM DTT; 0.5 mM PMSF, Phosphatase Inhibitor Cocktail 1:100; Protease Inhibitor Cocktail 1:200) and centrifuged at 1000×*g* for 10 min (4 °C)—P1 fraction. Separated supernatant was centrifuged at 14000×*g* for 20 min (4 °C) and S2 fraction (supernatant) was collected. The pellet, after buffer addition, was frozen as P2 fraction. Protein concentrations for both assays were performed using a BCA Protein Assay from Thermo Scientific (Pierce, Rockford, IL, USA).

The content of synaptic proteins was assessed by immunoblotting as previously described [30, 31]. Cortex homogenates/S2 fraction/P2 fraction were loaded with 15–30 μg of protein diluted in Laemli buffer per each tissue sample and loaded in 10% SDS-PAGE gels, then transferred on to PVDF membrane. The purity of S2 and P2 fractions was further analyzed by immunoblotting using antibodies against lactate dehydrogenase (LDH) and cadherin as cytosolic and membrane fraction markers, respectively. Western blot membranes were blocked in 5% milk and incubated overnight at 4 °C with antibodies (against nNOS, NR1, PSD-95, synaptophysin, synaptotagmin, syntaxin-1, Munc 18–1, vti1a) in recommended dilution in 1% milk and then for 1 h in 1% milk with secondary reagents. The protein bands were visualized with enhanced chemiluminescence, using G-BOX (Syngene). Data were expressed as fold change in fluorescent band intensity of target antibody divided by GAPDH, which is used as a loading control. The values of vehicle or control groups were used as a baseline and set to a relative protein expression value of 1. All band intensity quantifications were analyzed using GeneTools.

#### Statistical Analyses

An ANOVA followed by Dunnett post hoc test was used to detect intervals in which significant changes occurred for all data sets where a parameter was measured across time points within a treatment. Student’s *t* test or the Mann-Whitney *U* test was applied when two populations of responses were examined. Error bars represent the SD or SEM, which is specifically indicated, *p < 0.05, **p < 0.01, and ***p < 0.001.

## Results

### Characteristics of the AOM Model

Activity assessment revealed a difference between control and AOM-injected animals in the distance traveled following placing in a novel cage (Fig. 1a). Repeated measures ANOVA showed a strong effect of treatment [*F*(1,14) = 15.66, *p* < 0.01] and testing time [*F*(5,70) = 23.51, *p* < 0.001] as well as treatment × time effect [*F*(5,70) = 2.47, *p* < 0.05]. The activity of both groups gradually decreased; however, AOM mice started traveling shorter distances as early as 15–30 min postinjection, as demonstrated by planned comparisons (*p* < 0.01 for all time intervals after 0–15 min).

The neurological score at each time point was defined as the summation of six reflexes, and the average values can be seen in Fig. 1b. Continuous and significant neurological decline towards coma was observed starting from the sixth hour following AOM injection, as revealed by Friedman nonparametric analysis (*p* < 0.001) followed by individual Wilcoxon comparisons of dependent groups.

AOM injection led to a significant increase in the concentration of ammonia and inflammatory cytokines, TNF-α and IL-6, and activities of liver damage marker enzymes, ALT and AST in plasma (Table 1). In vivo ^1^H spectrometric analysis revealed ALF-specific changes including increase of Glu/Gln ratio and *myo*-inositol in cerebral cortex of AOM-treated mice (Fig. 2a). Extracellular Glu concentration, as determined in microdialysates from freely moving mice, started to increase on 17 h after AOM administration (Fig. 2b).

### Stage Dependence of Electrophysiological Responses of Cortical Slices from AOM Mice

#### FPs, PPF, and LTP

**Fig. 3:**
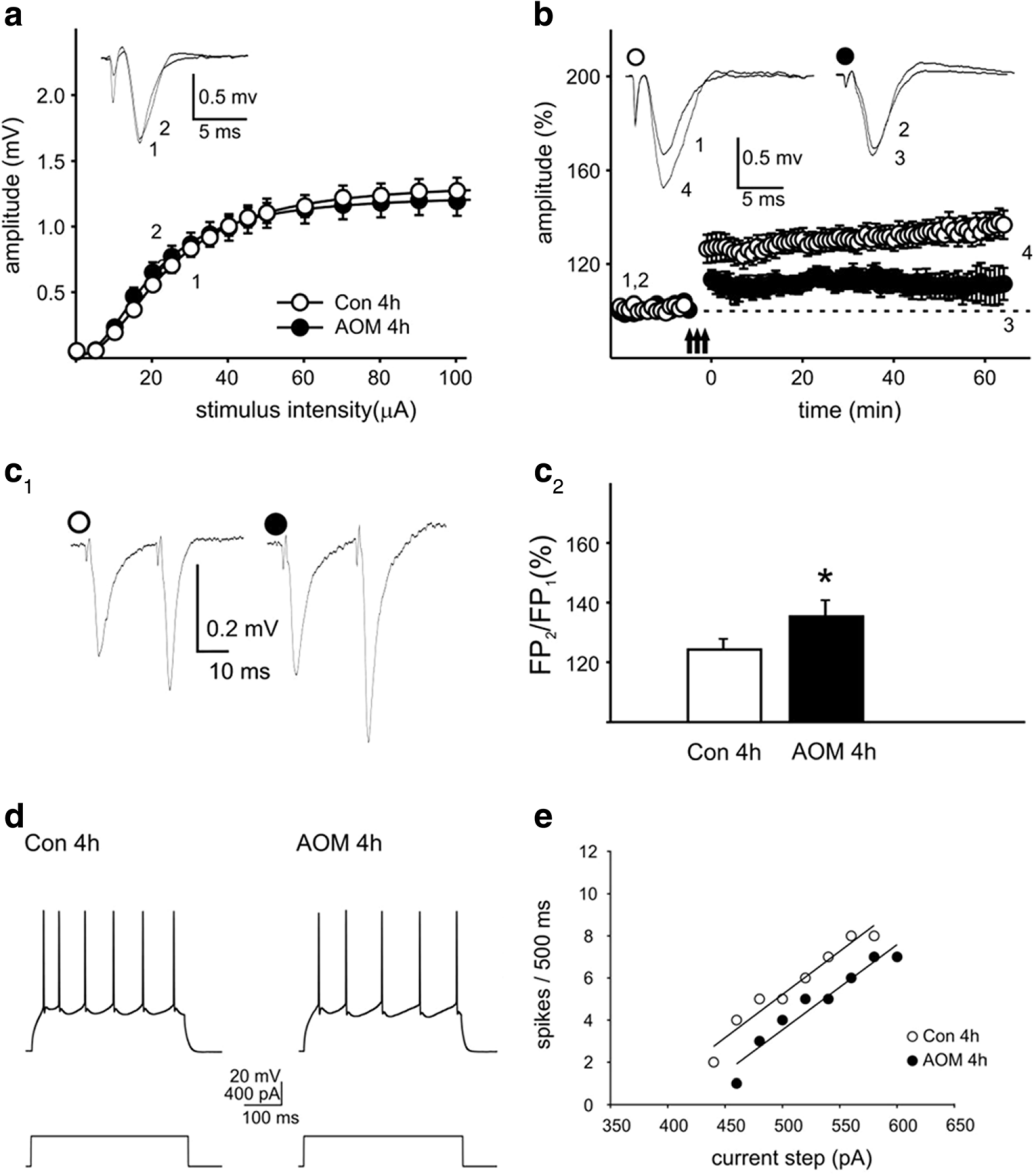

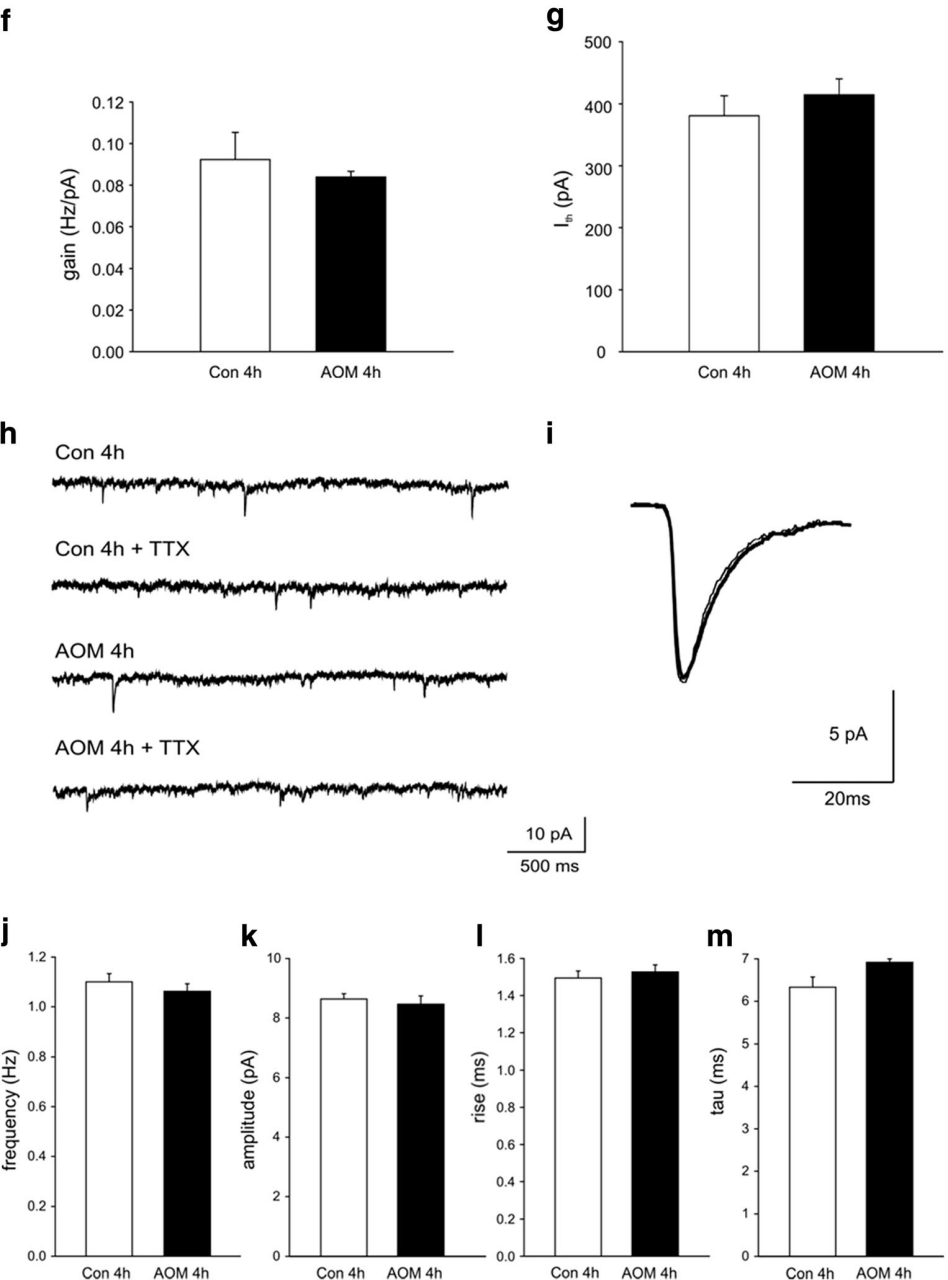
Electrophysiology of ALF-induced changes in cerebrocortical slices obtained from AOM mice at asymptomatic stage. **a**–**c2** The effects of AOM on FPs in slices prepared from asymptomatic mice (AOM 4 h). **a** The amplitudes of FPs of control (Con 4 h) and AOM mice (AOM 4 h). **b** Impairment of LTP. *Insets* in **a**, **b** show the superposition of averaged FPs recorded in representative experiments at times indicated by *numbers*. *Arrows* in **b** denote the TBS. **c1** Examples of individual FPs evoked by paired stimuli in slices from control (*open circles*) and AOM-treated mice (*filled circles*). **c2** Summary quantification of the average PPF ratio (±SEM); *p < 0.05. **d**–**g** In slices prepared from asymptomatic mice (AOM 4 h), the basic electrophysiological properties of layer II/III pyramidal neurons remain unchanged (apart from the resting membrane potential, see text). **d** An example of response of control pyramidal neuron (*left trace*) and response of a cell from AOM-treated (AOM 4 h) mouse (*right trace*) to a depolarizing current pulse (*lower traces*). **e** The injected current vs. spiking rate relationship in a cell originating from control mouse (*open circles*) and in a neuron originating from AOM-treated mouse (*filled circles*). *Solid lines* represent the linear fits to the experimental data. **f** The mean gain (±SEM) and **g** mean firing threshold (±SEM) of pyramidal neurons prepared from control (*white bars*; 18 cells; *n* = 5) and AOM-treated animals (*black bars*; 21 cells; *n* = 5). **h**–**m** Lack of changes in sEPSCs in slices from asymptomatic mice (AOM 4 h). **h** Examples of raw records from control neuron (*upper pair of traces*) and neuron from AOM mouse (*lower pair of traces*) recorded before (Con 4 h, AOM 4 h) and after addition of TTX (Con 4 h + TTX, AOM 4 h + TTX). **i** The superposition of averages of all individual sEPSCs detected during 4 min baseline recordings from control neuron (*thin line*) and a neuron originating from an AOM-treated mouse (*thick line*). *Bar graphs* illustrate a lack of the influence of AOM on **j** mean frequency, **k** mean amplitude, **l** mean rise time, and **m** mean decay time constant of sEPSCs. In **j**–**m**, neurons originating from control mice, *error bars* represent the SEM (*white bars*; 18 cells; *n* = 5) and AOM-treated animals (*black bars*; 21 cells; *n* = 5)

**Fig. 4:**
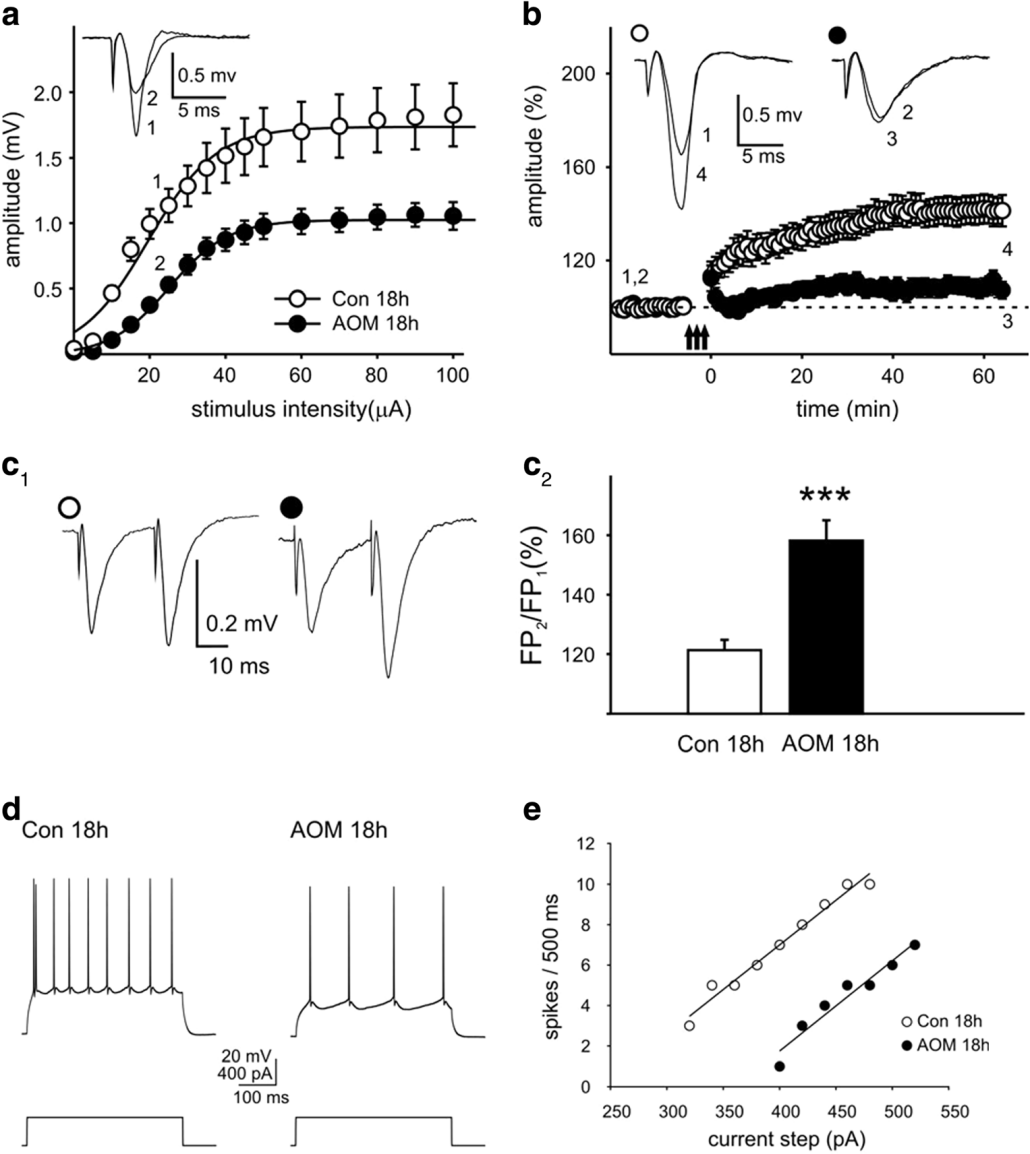

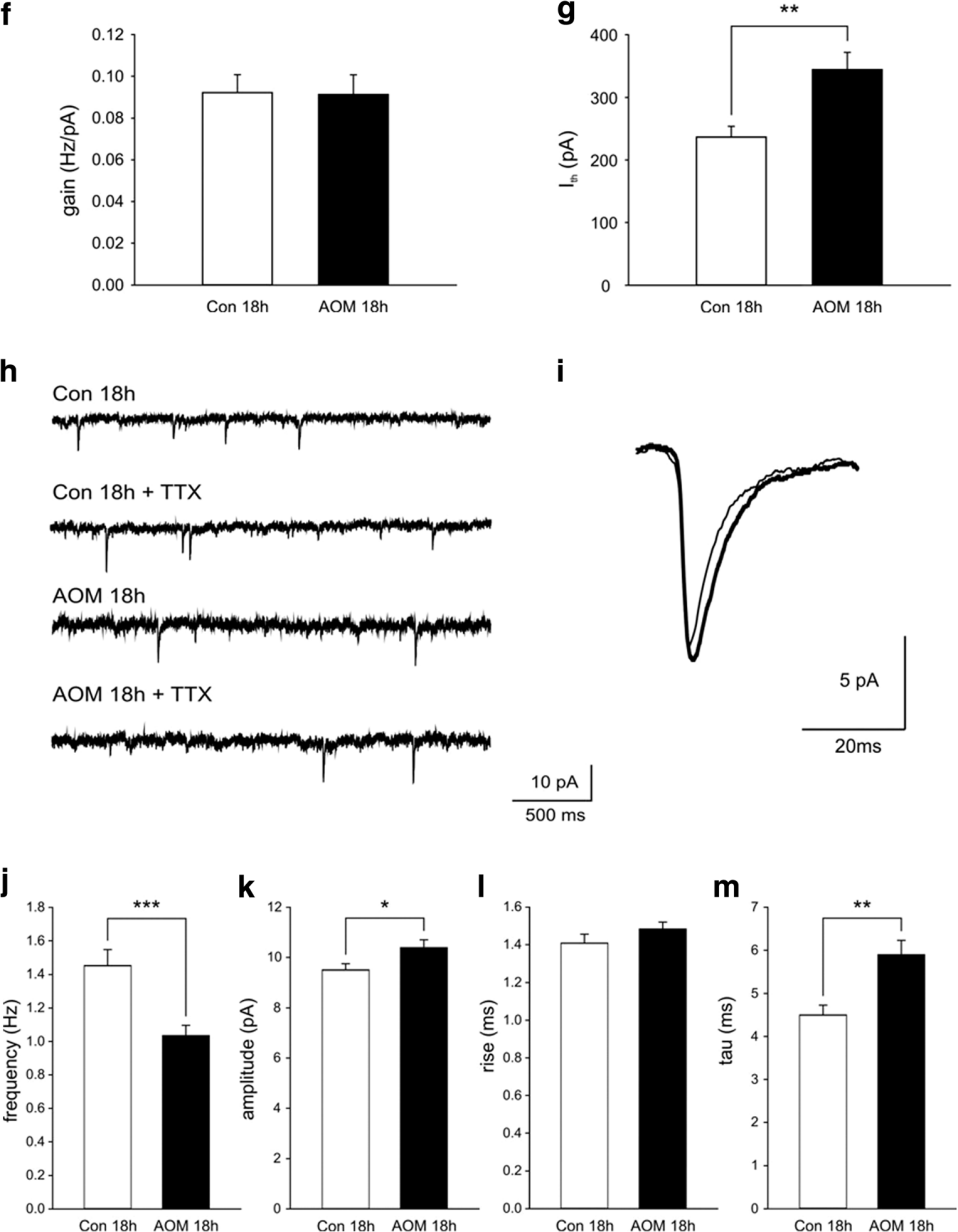
Electrophysiology of ALF-induced changes in cerebrocortical slices obtained from AOM mice at symptomatic stage. **a**–**c2** The effects of AOM on FPs in slices prepared from symptomatic mice (AOM 18 h). **a** The amplitudes of FPs of control (Con 18 h) and AOM mice (AOM 18 h). **b** Impairment of LTP. **c1** Examples of individual FPs evoked by paired stimuli in slices from control and AOM-treated mice. **c2** Summary quantification of the average PPF ratio (±SEM); ***p < 0.001. *Symbols* as in Fig. 3 **a**–**c2**. **d**–**g** In slices prepared from symptomatic mice (AOM 18 h), the excitability of layer II/III pyramidal neurons is reduced. **d** An example of response of control pyramidal neuron (Con 18 h) (*left trace*) and response of a cell from symptomatic mouse (AOM 18 h) (*right trace*) to a depolarizing current pulse (*lower traces*). **e** The injected current vs. spiking rate relationship in a cell from control mouse and in a neuron from AOM-treated mouse. **f** The mean gain (±SEM) and **g** mean firing threshold (±SEM) of pyramidal neurons prepared from control (23 cells; *n* = 6) and AOM-treated animals (21 cells; *n* = 6); **p < 0.01. *Symbols* as in Fig. 3 **d**–**g**. **h**–**m** sEPSCs in slices prepared from symptomatic rats (AOM 18 h). **h** Examples of raw records from a control neuron (*upper pair of traces*) and a neuron from AOM mouse (*lower pair of traces*) recorded before (Con 18 h, AOM 18 h) and after addition of TTX (Con 18 h + TTX, AOM 18 h + TTX). **i** The superposition of averages of all individual sEPSCs detected during 4-min baseline recordings from a control neuron (*thin line*) and a neuron originating from an AOM-treated mouse (*thick line*). *Bar graphs* illustrate the effect of AOM on **j** mean frequency, **k** mean amplitude, **l** mean rise time, and **m** mean decay time constant of sEPSCs. In **j**–**m**, neurons originating from control mice, *error bars* represent the SEM (*white bars*; 23 cells; *n* = 6) and AOM-treated animals (*black bars*; 21 cells; *n* = 6); *p < 0.05, **p < 0.01, ***p < 0.001

Analyses of FPs revealed no significant differences in the stimulus-response relationship between slices prepared 4 h after AOM administration (AOM 4 h) and control preparations (Fig. 3a). In contrast, in brain slices prepared from mice with neurological decline (AOM 18 h), the amplitude of FPs was markedly lowered over a wide range of stimulation intensities (Fig. 4b). Parameters characterizing input-output curves of FPs, calculated using the Boltzmann fits, are summarized in Table 2. The mean amplitude of FPs, measured 60–75 min after delivery of TBS, to induce LTP, was 128.6 ± 4.2% of baseline in the control asymptomatic group (Fig. 3b) and 141.1 ± 6.1% of in the Con 18-h group (Fig. 4b). LTP was significantly attenuated in the slices obtained from AOM-treated animals both in asymptomatic stage (108.5 ± 6.2%; *p* < 0.01 vs. Con 4 h; Fig. 3b) and in symptomatic stage (108.4 ± 3.6%; *p* < 0.001 vs. Con 18 h group; Fig. 4b). Despite a lack of changes in the amplitudes of FPs, in slices obtained from AOM mice at asymptomatic stage, PPF ratio was significantly increased (Con 4 h 117.6 ± 5.1% vs. AOM 4 h 130.9 ± 4.4%; *p* < 0.05; Fig. 3c1, c2). PPF was further increased in slices obtained 18 h after AOM administration (Con 18 h 121.5 ± 3.5% vs. AOM 18 h 158.3 ± 6.9; *p* < 0.001; Fig. 4c1, c2).

**Table 2:** Effects of the treatment with AOM on parameters characterizing stimulus-response curves of field potentials

	*V*_max_ (Mv)	*U*_*h*_ (μA)	*S*	Number
Con 4 h	1.19 ± 0.40	23.86 ± 3.20	9.16 ± 4.21	11
AOM 4 h	1.13 ± 0.39	22.77 ± 4.50	8.14 ± 4.10	12
Con 18 h	1.80 ± 0.40	19.38 ± 6.20	8.20 ± 3.59	18
AOM 18 h	1.05 ± 0.36*	27.77 ± 5.04*	7.59 ± 3.10	25

*p < 0.001; AOM 18 h vs. control animals (Con 18 h)

#### Pyramidal Neuron Membrane Excitability

Whole-cell recordings were obtained from layer II/III neurons exhibiting a regular spiking firing pattern in response to a depolarizing current pulse (Figs. 3d and 4d). In slices prepared 4 h after AOM administration, the resting membrane potential of neurons was significantly different from control neurons (−78.1 ± 0.8 vs. −73.6 ± 0.6 mV, respectively; *p* < 0.001). A similar difference existed between neurons in slices prepared 18 h after AOM injection and control cells (−78.6 ± 2.0 vs. −73.4 ± 0.7 mV, respectively; *p* < 0.05). The input resistance of neurons originating from AOM 4 h mice was not different from that of the control group (143.5 ± 2.8 vs. 151.1 ± 4.7 MΩ, respectively; *p* = 0.36); however, the input resistance of neurons originating from AOM 18-h mice was significantly lower from that of the respective control group (123.4 ± 11.6 vs. 167.1 ± 12.0 MΩ, respectively; *p* < 0.01).

Analyses of the relationship between the injected current and the firing rate (gain; Figs. 3e and 4e) demonstrated that AOM did not modify the intrinsic excitability of pyramidal neurons. Neither in slices prepared 4 nor 18 h after AOM administration, the differences between experimental and control mice were significant (Figs. 3f and 4f). However, in slices obtained 18 h after AOM administration, the calculated threshold current for the generation of the action potential was significantly higher than in the respective control mice (Con 18 h; 344.0 ± 27.7 vs. 236.6 ± 17.2 pA; *p* < 0.01; Fig. 4g). This effect appears to be related to a markedly lower membrane resistance of neurons in the AOM 18-h group. In contrast, the average spike threshold current in the AOM 4-h mice was not different from the Con 4-h mice (Fig. 3g).

#### Excitatory Postsynaptic Currents

In the cells originating from the AOM 4-h mice, the mean frequency of sEPSCs was similar to respective control (Fig. 3j). Similarly, the mean amplitudes of sEPSCs in the AOM 4-h mice and in the respective control were not significantly different (Fig. 3k). However, in the cells originating from AOM 18-h mice, the mean frequency of sEPSCs was significantly lower in comparison to that in the cells from control animals (1.0 ± 0.1 vs. 1.5 ± 0.1 Hz; *p* < 0.001; Fig. 4j). AOM, acting for 18 h, also increased the mean amplitude of sEPSCs (10.4 ± 0.3 vs. 9.5 ± 0.2 pA; *p* < 0.05; Fig. 4k), and additionally, it significantly increased the mean decay time constant of sEPSCs (from 4.5 ± 0.2 to 5.9 ± 0.3 ms; *p* < 0.001; Fig. 4m). AOM did not alter the mean rise time of sEPSCs either in the AOM 4-h mice (Fig. 3l) or in the AOM 18-h mice (Fig. 4l).

The effect of Na^+^ channel blockade on recorded EPSCs was investigated in separate samples of neurons obtained from control and AOM-treated mice (Con 4 h vs. AOM 4 h and Con 18 h vs. AOM 18 h). The addition of 1 μM TTX to ACSF did not result in significant changes of the mean frequency, mean amplitude, mean rise, and mean decay time constant of sEPSCs (Table 3) either in control or in experimental groups. This finding indicates that in our whole-cell recordings, the contribution of action potential-induced release of neurotransmitter from presynaptic terminals is negligible.

**Table 3:** Comparison of sEPSC and mEPSC parameters in control and AOM-treated (4 and 18 h) mice

	Con 4 h		Con 18 h	
	sEPSCs	mEPSCs	sEPSCs	mEPSCs
Frequency (Hz)	1.1 ± 0.1	1.1 ± 0.1	1.3 ± 0.1	1.3 ± 0.1
Amplitude (pA)	8.4 ± 0.2	8.4 ± 0.2	9.4 ± 0.2	9.3 ± 0.2
Rise (ms)	1.5 ± 0.1	1.5 ± 0.1	1.4 ± 0.0	1.4 ± 0.0
Tau (ms)	6.2 ± 0.3	6.2 ± 0.3	4.6 ± 0.1	4.6 ± 0.1
	AOM 4 h		AOM 18 h	
	sEPSCs	mEPSCs	sEPSCs	mEPSCs
Frequency (Hz)	1.1 ± 0.1	1.0 ± 0.1	1.0 ± 0.0	1.0 ± 0.0
Amplitude (pA)	8.9 ± 0.4	8.8 ± 0.4	10.3 ± 0.2	10.2 ± 0.2
Rise (ms)	1.5 ± 0.1	1.5 ± 0.1	1.5 ± 0.1	1.5 ± 0.1
Tau (ms)	6.8 ± 0.2	6.8 ± 0.2	5.9 ± 0.1	5.9 ± 0.1

Data are presented as the mean ± SEM

#### Ultrastructural Analysis of Cortical Synapses

**Fig. 5:**
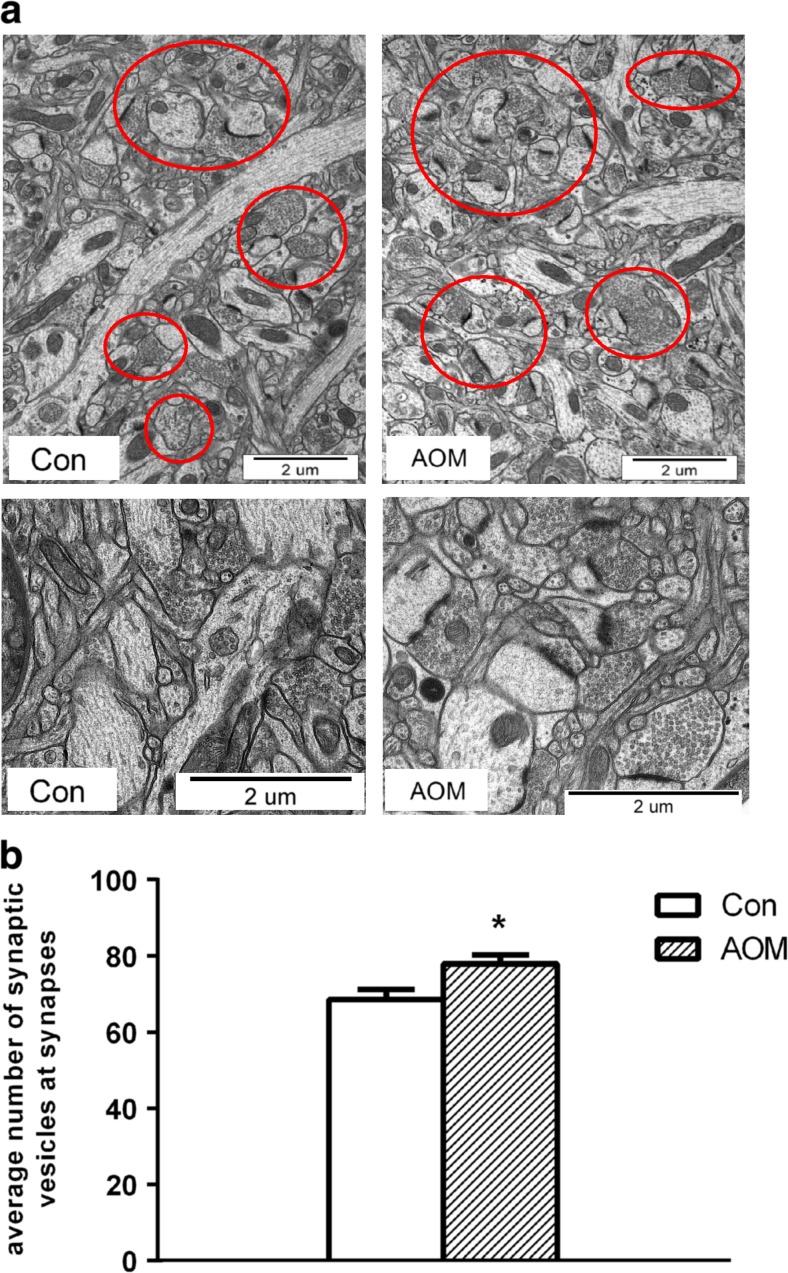
Electron microscopy of the cerebral cortex of control and AOM-treated mice at symptomatic stage. **a** Representative synapses are indicated by *red circles*. **b** Average number of synaptic vesicles at synapses counted from 50 randomly selected synapses of control (*n* = 3) and AOM-treated mice (*n* = 4); *asterisk* indicates *p* < 0.05 vs. control animals (Con). Results are means ± SEM

Electron microscopy analysis of the cortex from the symptomatic AOM mice showed an increased number of synapses showing abundance of sv in the presynaptic zone. The numbers of sv in randomly selected sections from symptomatic mice were (~15%) higher than in the control (Fig. 5a, b).

### Expression of Synaptic Proteins at Different Stages of ALF

#### Presynaptic Proteins

**Fig. 6:**
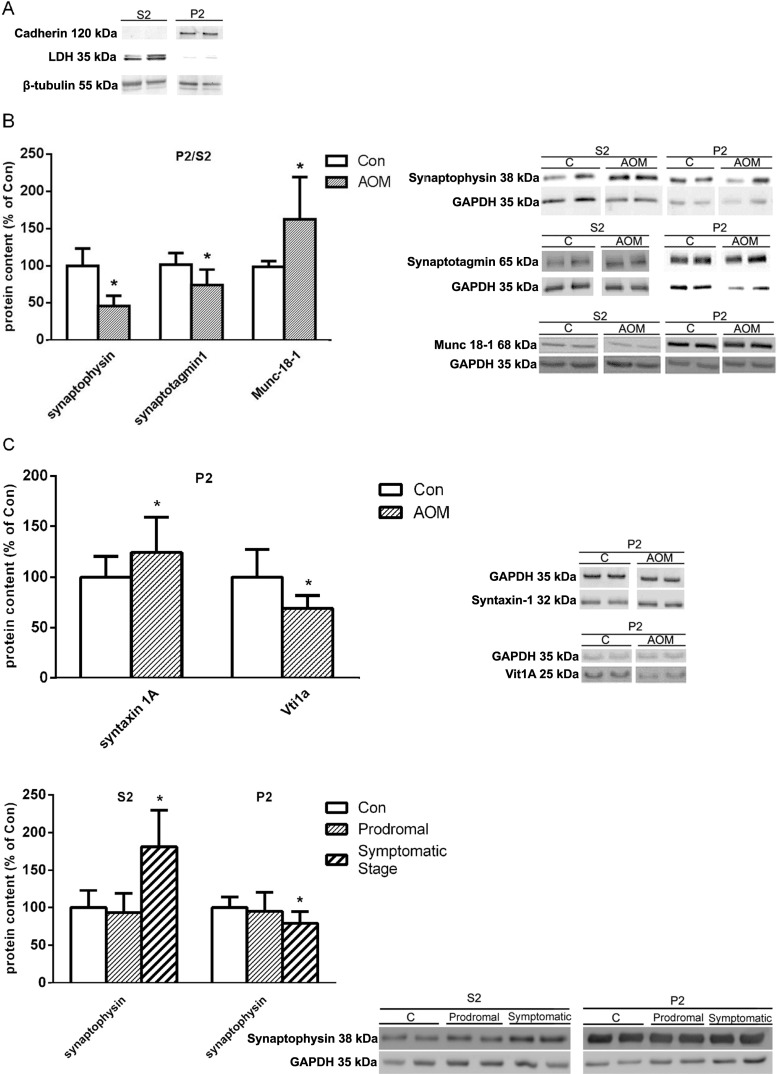
Protein content of selected presynaptic proteins. **a** Purity of S2 and P2 fraction. **b** Synaptophysin, synaptotagmin-1, and Munc 18–1 protein contents in the cerebral cortex of control and symptomatic AOM-treated mice shown as membrane (P2) to cytosolic (S2) fraction ratio (P2/S2) (*n* = 8), followed by representative electrophorograms. **c** Syntaxin-1 and vti1A protein contents in membrane fraction (P2) in control and symptomatic AOM-injected mice (*n* = 8), followed by representative electrophorograms. **d** Changes in synaptophysin protein content in cytosolic (S2) and membrane fraction (P2) at prodromal and symptomatic stage of ALF (*n* = 6), followed by representative electrophorograms. *Asterisk* indicates *p* < 0.05 vs. control animals (Con). Results are means ± SEM

The membrane to cytoplasm content ratio (P2/S2) of presynaptic proteins, synaptophysin and synaptotagmin, were decreased by ~50 and ~30%, respectively, in the frontal cortex of symptomatic mice as compared to the control and that of Munc 18–1 was increased by ~70% (Fig. 6b). The P2 fraction content of syntaxin-1 revealed an ~20% increase, and the level of vti1a protein, which characterizes vesicles driving spontaneous release, was significantly decreased (Fig. 6c). In the AOM mice at the asymptomatic stage, neither the S2 nor the P2 content of synaptophysin was changed (Fig. 6d). The S2 protein content of synaptophysin increased gradually between asymptomatic and symptomatic stages (Fig. 6d). The cytosolic and membrane fraction markers LDH and cadherin were analyzed by immunoblotting and confirmed a purity of both fractions (Fig. 6a).

#### Postsynaptic Proteins

**Fig. 7:**
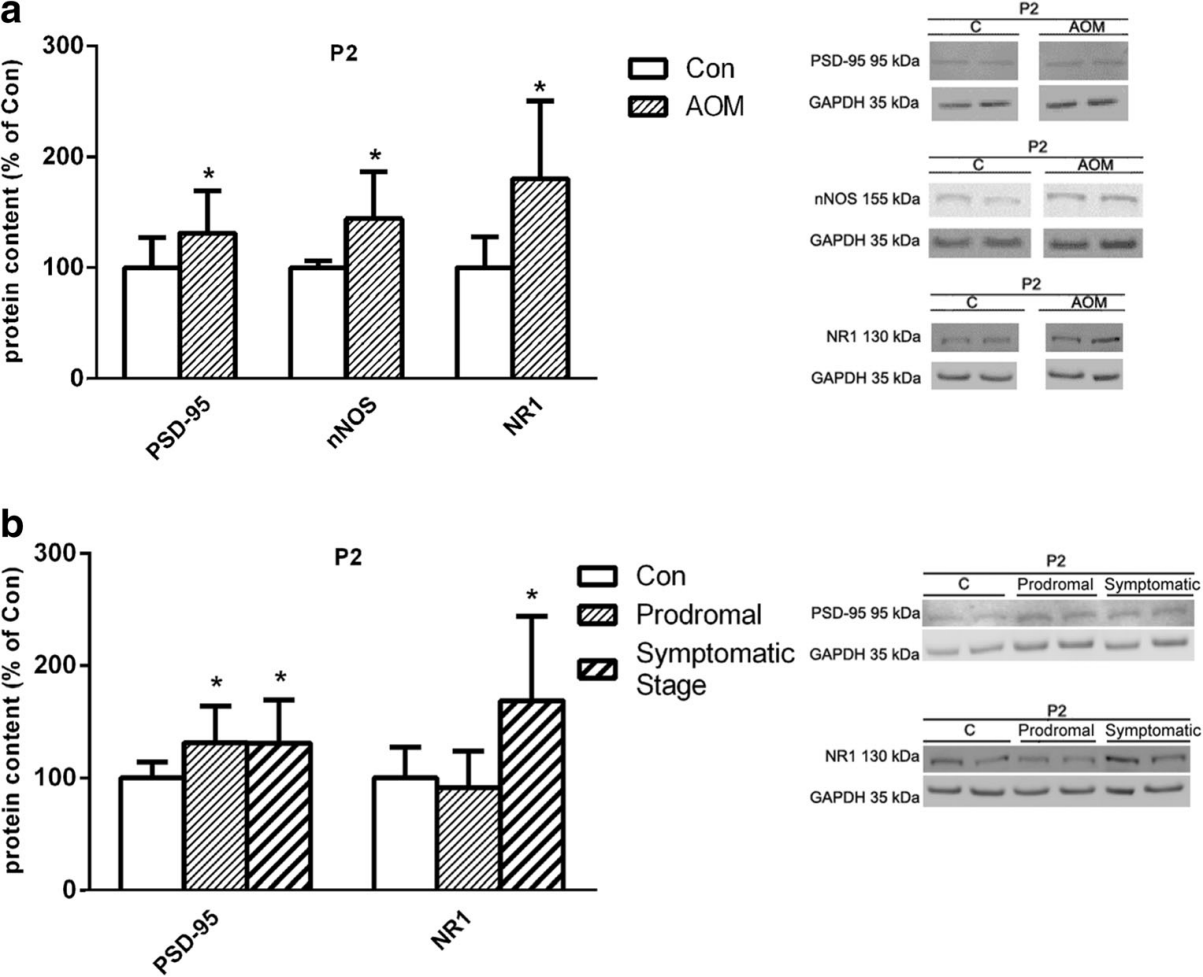
Protein content of selected postsynaptic proteins. **a** PSD-95, nNOS, and NR1 protein contents in membrane fraction (P2) in control and symptomatic AOM-treated mice (*n* = 8), followed by representative electrophorograms. **b** Changes in PSD-95 and NR1 protein content in membrane fraction (P2) at prodromal and symptomatic stage of ALF (*n* = 6), followed by representative electrophorograms. *Asterisk* indicates *p* < 0.05 vs. control animals (Con). Results are means ± SEM

Analysis of postsynaptic complex NMDAR/PSD-95/nNOS revealed ~16, ~40, and ~35% increase of its particular components, i.e., NR1 subunit, PSD-95, and nNOS protein, respectively, in P2 fraction from the frontal cortex of symptomatic AOM-treated mice (Fig. 7a). Moreover, in the prodromal stage of AOM, the PSD-95, but not NR1 proteins level, was increased (Fig. 7b).

## Discussion

The present study provided an exhaustive description of alterations in synaptic transmission, ultrastructure, and expression of synaptic transmission-related proteins in the frontal cortex of mice with ALF. In our hands, the AOM model reproduced a wide spectrum of changes in blood and brain biochemistry as well as in neurophysiological and behavioral manifestations of ALF, each reflecting those reported for acute HE in different animal ALF models [25, 32–34] and the health status of patients with ALF [35–37]. Progression of neurological deficit in AOM-treated mice suggested that ALF could affect and/or modify information processing in mice frontal cortex and prompted us to carry out detailed investigations of electrophysiological properties of pyramidal neurons and excitatory synapses at the asymptomatic and symptomatic stage. The results documented that disturbances in neurotransmission coincide with impaired neurological status of the animals. At the asymptomatic stage, no significant changes in most parameters characterizing synaptic transmission, such as FP amplitude and the frequency and amplitude of sEPSCs/mEPSCs, were evident. However, despite lack of those changes, the PPF ratio was elevated at this stage. As in PPF, an increase in the amplitude of the response to the second pulse of a pair is determined by a presynaptic mechanism involving the residual calcium signal arising from calcium entry through voltage-gated calcium channels [38]; this result may indicate that early effects of ALF, at asymptomatic stage, include disturbances in calcium-buffering mechanisms within presynaptic terminals. At the level of neurological status, the PPF ratio was higher than at the asymptomatic stage; however, this effect was accompanied by other marked changes including a reduction in the frequency of sEPSCs/mEPSCs.

As the number of glutamatergic synapses in the frontal cortex of AOM-treated mice appeared unchanged, the observed decrease in sEPSC/mEPSC frequency (Fig. 4j) was most likely due to a decreased probability of release of glutamate quanta from presynaptic terminals, which may also underlie a decrease in the amplitude of FPs. This conclusion is further supported by the finding that the distribution of synaptophysin, synaptotagmin, and vti1a proteins was significantly distorted in the symptomatic AOM-injected mice.

At the level of neurological status, however, an increased amplitude and a longer decay time constant of sEPSCs/mEPSCs were also evident. These changes appear to be of postsynaptic origin, related to the reactivity and kinetics of ionotropic glutamate receptors and may be related to observed changes in the postsynaptic complex proteins. Moreover, in the symptomatic AOM-treated mice, a markedly lower membrane resistance of neurons was evident. These effects were not related to a direct action of ammonium ion on neurons [8] as the slices had been incubated for several hours in the ACSF before recordings began.

Quantification of sv and analysis of changes in the distribution of synaptic proteins further confirmed the contribution of presynaptic modifications to ALF-induced changes in neurotransmission in the model. The number of sv per synapse was found increased, which is consistent with the decrease of membrane-to synaptoplasm content ratio of synaptophysin and synaptotagmin, suggesting a decreased efficiency of vesicle trafficking to the membrane. It is tempting to speculate that increased association with the membranes of the fusion protein Munc-18-1 and the docking protein syntaxin-1 may reflect their response of the deficient supply of vesicles for interaction with these proteins. However, analysis of the expression and positioning of many other components of the docking machinery, not considered here, including Munc-13, calcium-dependent activator protein for secretion, SNAP-25, and synaptobrevin-2 are needed to verify this speculation. The vps10p tail interactor 1a (vti1a) protein, acting together with vesicle-associated membrane protein 7, characterizes vesicles driving spontaneous release [11]. Since the postsynaptic currents measured corresponded to mEPSCs, a product of activity-independent, spontaneous release of glutamate from presynaptic terminals, the ALF-related decrease of vti1a expression may therefore be considered as a likely, albeit perhaps not the only cause of the inhibition of mEPSCs.

In our study, slices from AOM-treated mice with early, minor neurological deficits and from the symptomatic animals showed impaired LTP. Impairment of LTP has been repeatedly demonstrated in chronic liver failure [4, 39], the studies mostly focusing on the role of modulation of the NMDA-sGC-nNOS-NO-cGMP pathway [5, 40, 41]. The present study documents, to our knowledge for the first time, that although the expression of proteins critical for postsynaptic activity (the NR1/PSD-95/nNOS complex) was increased, LTP was nevertheless impaired. Thus, mechanisms operating beyond that complex appear to underlie the observed effect. It is conceivable that they may involve observed increase in the levels of proinflammatory cytokines, IL-6 and TNF-α, as activation of the cytokine network in the brain has been shown to be involved in LTP [42].

In conclusion, the here presented data demonstrate that symptomatic ALF is associated with decreased synaptic transmission which appears to be related to derangements of presynaptic proteins, resulting in inefficient sv docking to the synaptic membrane. By contrast, the content of postsynaptic proteins is enhanced, which may reflect a response to decreased presynaptic activity. Evidently, the postsynaptic modifications, occurring during symptomatic ALF, appear insufficient to provide effective compensation for the decline of LTP.

### Authors’ Contributions

MZ/JA conceived the idea, designed the protocol, and wrote the manuscript.

MP/BB/JS/MF-B/RP performed all the analyses for the study.

RKF/GH/BZ helped write the manuscript and intellectually refine the protocol.The authors want to give special thanks to the Small Animal Magnetic Resonance Laboratory team for help with carrying out the MRI measurements.
